# Reforming medical career progression: a call for merit-based systems

**DOI:** 10.3389/fmed.2025.1643399

**Published:** 2025-12-01

**Authors:** Francesco Maria Bulletti, Maurizio Guido, Maria Elisabetta Coccia, Antonio Palagiano, Evaldo Giacomucci, Carlo Bulletti

**Affiliations:** 1 Department of Maternity and Gynaecology, CHUV, Lausanne, Switzerland; 2 Department of Obstetrics and Gynaecology, University of Calabria, Cosenza, Italy; 3 Department of Biomedical, Experimental and Clinical Sciences “Mario Serio”, University of Florence, AOU Careggi, Florence, Italy; 4 Fertility and Sterility Centre (CFA), Naples, Italy; 5 Obstetrics and Gynaecology Unit, Ospedale Maggiore, Bologna, Italy; 6 Department of Obstetrics, Gynaecology and Reproductive Sciences, Yale University, New Haven, CT, United States

**Keywords:** medical career, competence, meritocracy, professional value score, innovation score

## Abstract

**Objective:**

To identify and address systemic barriers undermining the meritocratic advancement of medical professionals in Italy and to propose a transparent, performance-driven recruitment model.

**Study design:**

A critical narrative review and conceptual framework proposal supported by an analysis of current systemic limitations and international benchmarking data.

**Methods:**

We conducted a narrative review involving structured searches of international and Italian sources, followed by thematic synthesis and the development of two merit frameworks—Merit-based Professional Value Score (MPVS) and Integrity and Impact Score (IIS)—featuring standardized indicators and peer-normalized scoring metrics.

**Results:**

Italy's medical system, despite high economic capacity, underperforms due to persistent non-meritocratic structures. Key challenges include political interference in residency selection, low return rates of expatriated physicians (>11,000 currently practicing abroad), and biased hiring mechanisms. Women and internationally trained candidates encounter disproportionate barriers. Across medical systems, output-only metrics (e.g., H-index) has proven insufficient. We propose MPVS and IIS as transparent, auditable tools that integrate risk-adjusted outcomes, patient safety indicators, patient-reported measures, teaching, research, and integrity domains. A worked example illustrates end-to-end scoring process and decision thresholds. Furthermore, a new protocol is proposed featuring anonymized candidate evaluation based on two metrics:

- *Medical Professional Value Score (MPVS)*—integrates clinical outcomes, teaching performance, and professional conduct.
- *Impact Innovation Score (IIS)*—evaluates research relevance, innovation capacity, and applied contributions. Cross-linked digital verification, external audits, and rotating blinded selection panels under national anti-corruption oversight form the governance backbone.

**Conclusion:**

Implementing this model would help reverse Italy's brain drain, restore merit-based standards in healthcare sector, and provide a replicable framework for other health systems pursuing transparency, quality, and equity.

## Highlights

- Italy's physician-selection system is hampered by nepotism, opaque metrics, and political interference, resulting in talent loss and diminishing quality of care.
- Mandate a digital performance registry documenting audited clinical outcomes, teaching metrics, and research impact, appended to every applicant's CV.
- Implement two composite indicators—MPVS and IIS—to quantify competence and real-world innovation, using these scores as the basis for all short-listing on these scores.
- Employ rotating, anonymized selection committees and publish final score sheets to deter patronage and “tailor-made” job calls.
- Empower ANAC and the Court of Auditors to invalidate appointments and sanction false declarations.
- Together, these measures would enhance patient outcomes, retain high-performing clinicians, and rebuild public trust in Italy's healthcare institutions.

## Introduction

Healthcare quality is routinely benchmarked internationally using a spectrum of indicators, ranging from simple measures—such as the proportion of physicians who report job satisfaction or the percentage of patients who rate their care highly—to more complex metrics, including hospital-bed utilization, staffing ratios, and population-level outcomes (1–3). Recent political attacks on the public-health system (4, 5) and the rapid expansion of private-equity ownership in healthcare (6–9) have disrupted this core mission, often subordinating patients' interests to alternative objectives. Although Italy—used here as a test case—has a gross domestic product theoretically capable of sustaining robust health services (10–13), it persistently underperforms on these benchmarks (1–3) and has failed to increase its investment in the sector (14).

One important driver of Italy's lagging healthcare performance is the persistence of non-meritocratic practices (15–23) in the selection, recruitment, and promotion of medical personnel (15–25). The link between these practices and measurable health-system outcomes is complex (26, 27), yet their cumulative effect is unambiguous: Talented clinicians are discouraged, while patronage networks flourish. Efforts to train physicians in clinical algorithms and artificial-intelligence tools represent a positive step forward (28, 29), but embedding merit-based criteria into hiring and career advancement remains an urgent, unmet need (30).

**Figure 1:**
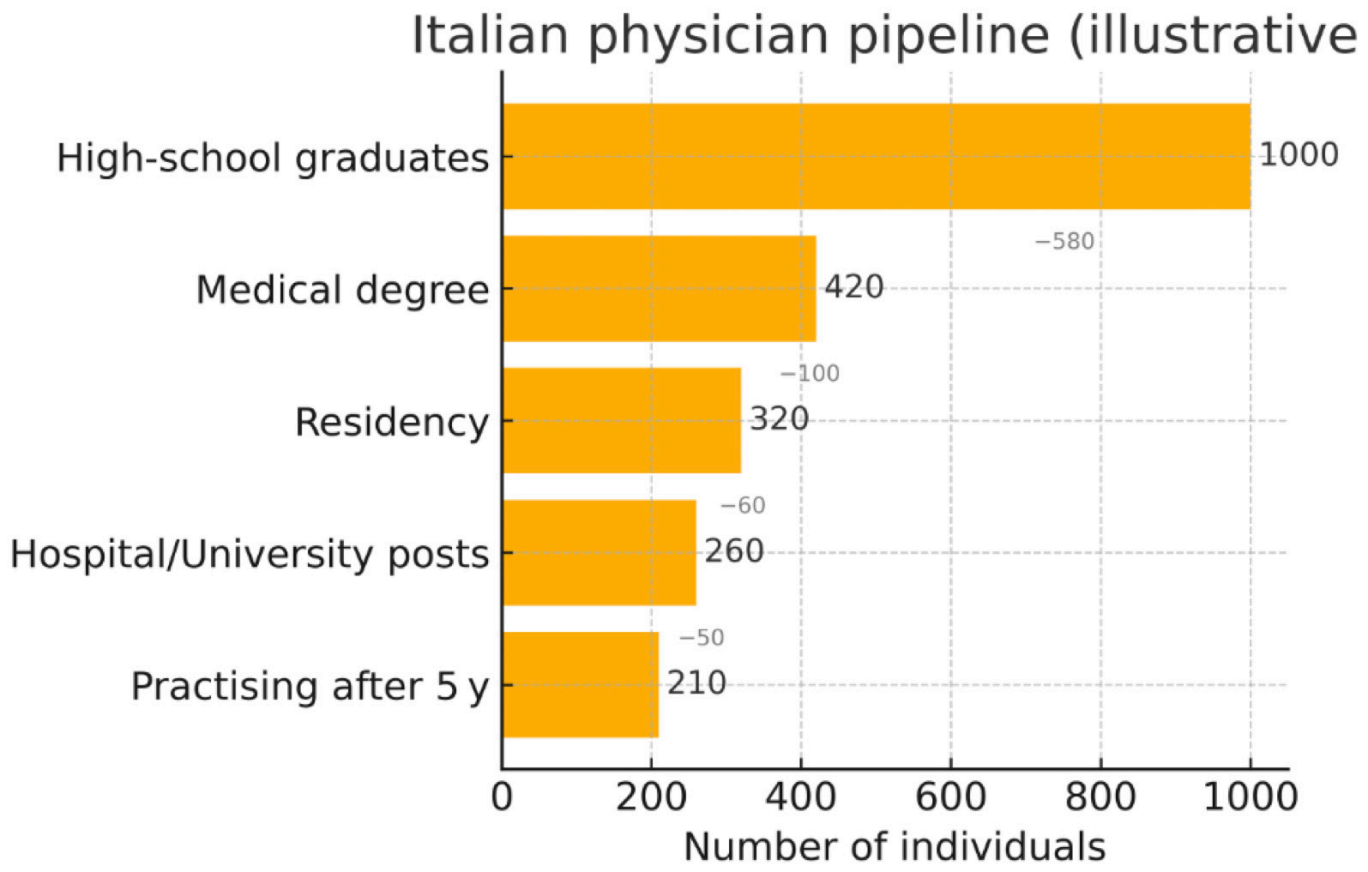
Flow diagram of the Italian physician pipeline, showing attrition at each career stage.

Italy's challenges originate in the educational pipeline. National and international standardized assessments (31–33) indicate declining performance in primary and secondary schools, a problem exacerbated by loosely regulated private institutions that award diplomas with minimal academic rigor. Although the Ministry of Education—recently rebranded the Ministry of Merit—has announced higher standards, one-third of Italians still struggle with basic reading comprehension and fewer than 5% reach full proficiency (Figure 1) (31–33). In this environment, many newly qualified doctors—and even experienced practitioners—emigrate in search of systems that genuinely reward competence and innovation (11–13, 15–30, 34, 35).

Recent data reveal a striking asymmetry between Italian physicians who emigrate and those who return. By 2021, more than 11,000 Italian doctors were practicing abroad in Organization for Economic Co-operation and Development (OECD) countries, W6 signaling a persistent “brain drain” that weakens the domestic workforce. The exact scale is uncertain—EU modeling suggests approximately 1,000 departures annually, W5 a figure many observers consider conservative. Although some clinicians eventually return with valuable international experience, the net loss remains considerable, especially in underserved areas and specialized fields.

In 2022, a total of 69,279 new physicians graduated across the European Union, corresponding to 155 medical degrees per 100,000 inhabitants. Italy slightly exceeds this average, with 166 medical degrees per 100,000 people (36). However, in the absence of ongoing migration, the real concern is not a shortage of physicians overall but an insufficient influx of younger doctors entering the workforce.

The fundamental issue is not a shortage of specialists *per se*, but Italy's struggle to attract foreign specialists (28–30). Working conditions in Italy are often inadequate, with low pay, limited job stability, and weak career prospects. Political influences on healthcare, along with well-publicized fraudulent recruitment (“Concorsi Truccati”), erode trust and deter skilled professionals. Meanwhile, other countries offer more appealing prospects, and both Italian and international physicians—especially those with strong qualifications and innovative ideas—would consider practicing in Italy if circumstances improved (28–30).

This imbalance is particularly pressing given Italy's aging population and escalating healthcare demands. Contributing factors include insufficient residency positions, non-meritocratic hiring, suboptimal working conditions, and inadequate financial incentives—each of which hampers the retention and repatriation of medical professionals (24–28). Addressing these challenges requires policy reforms that enhance training, advance careers, and encourage expatriate physicians to return, thereby strengthening the national healthcare system (27).

A comprehensive strategy must focus on enhancing educational standards, instituting transparent recruitment processes, and offering competitive remuneration. Only through such measures can Italy stem the outflow of medical talent and meet its expanding medical needs.

To provide a comprehensive overview of the evolving debate surrounding academic metrics and propose a conceptual framework for merit-based evaluation criteria in medical career advancement, this article explores the structural weaknesses in Italy's healthcare and academic systems, demonstrating how nepotism, superficial metrics, and subjective assessments undermine true merit. Building on national and international evidence, we propose reforms designed to foster a culture of transparency, accountability, and excellence.

## Method: search strategy and selection criteria

### Structure

- *Search strategy*—databases, gray literature, and policy docs; dates; and keywords.
- *Inclusion criteria*—jurisdiction, professional group, metric type, and language.
- *Data extraction*—variables, calibration rules, and double extraction.
- *Thematic synthesis***—**coding approach and triangulation.
- *Conceptual framework (MPVS and IIS)***—**domains, indicators, and weights.
- *Standardization and statistics*—peer-group normalization (0–100), risk adjustment, handling of small volumes, and missingness.
- *Sensitivity analyses*—domain reweighting ±10% and alternative equity indicators.

#### Conceptual framework

- *MPVS (Merit-Based Professional Value Score)*: A composite score of professional value across seven domains, with weighted contributions as follows: (1) Clinical effectiveness—risk-adjusted outcomes (30%); (2) Safety—complications and readmissions (20%); (3) Patient-reported outcomes/experience (15%); (4) Efficiency/throughput—case-mix adjusted (10%); (5) Teaching and mentorship (10%); (6) Research and innovation (10%); and (7) Service/leadership and quality improvement (QI) (5%).
- *IIS (Integrity and Impact Score)*. Measures governance and societal value across five domains, weighted as follows: (1) Transparency and conflict-of-interest (COI) compliance (25%); (2) Data completeness and audit pass rate (25%); (3) Equity and access—wait-time parity and underserved coverage (20%); (4) Professional conduct—substantiated complaints/discipline (15%); and (5) Continuous learning/CME (15%).
- *Scoring and normalization*. For each indicator *k*, raw values are risk-adjusted and peer-normalized within specialty and career stage over a 3-year rolling window, rescaled to 0–100 (higher is better). Domain score = mean of its indicators. Framework score = Σ(weight_*i*_ × domain_*i*_). Merit tier (illustrative thresholds): ≥85 “Excellent”; 75–84 “Strong”; 65–74 “Meets standard”; and < 65 “Needs development”. Guardrails: minimum patient volume, uncertainty bands, and publication of confidence intervals.

***Study Design:*** This study was conducted as a policy and practice review, providing a comprehensive and balanced overview of medical career recruitment and progression, regulatory frameworks, and existing guidelines, culminating in the proposal of a conceptual framework for reform. Its aim was to examine the limitations of widely used academic metrics—particularly the H-index—in evaluating merit within medical and academic career trajectories and to outline a performance-based, competency-driven model for promotion and recognition.

***Literature Search:*** A targeted search of the literature was carried out using PubMed, Scopus, Web of Science, and Google Scholar to identify relevant articles published between January 2000 and March 2025. The search focused on studies examining medical workforce recruitment, meritocracy, physician migration, or health-system governance in Italy or other high-income countries. The search strategy included keywords and Boolean operators such as: (“H-index” OR “bibliometrics”) AND (“academic promotion” OR “career evaluation” OR “scientific merit” OR “medical career”) AND (“research quality” OR “innovation” OR “authorship”) (“medical staff” OR physician^*^ OR doctor^*^) AND (recruit OR hir OR promotion OR career advancement) AND (merit OR meritocrat OR nepotism OR favoritism) AND (Italy OR Europe OR OECD).

***Inclusion Criteria:*** Publications were included if they addressed one or more of the following: (a) conceptual or empirical critiques of the H-index or similar metrics; (b) discussions of bias or inflation in academic publishing; (c) proposals for merit-based reforms in evaluation systems. Eligible sources included meta-research studies, policy documents, editorials, commentaries, and reviews focused on medical and life sciences disciplines. Searches were restricted to English or Italian publications, without study-design limitations. Reference lists of key papers were hand-screened, and relevant gray literature was incorporated from official Italian gazettes, EU documents, WHO reports, and major newspapers using custom Google domain searches (site:gazzettaufficiale.it, site:ec.europa.eu, site:who.int, site:repubblica.it, corriere.it, ilfattoquotidiano.it). Non-English articles and studies unrelated to the biomedical academic context were excluded.

***Data Extraction and Thematic Synthesis:*** Relevant data were extracted manually, including publication type, domain of focus, critique of current metrics, and suggested alternatives. A thematic synthesis approach was applied, organizing findings into three core categories: (1) erosion of the H-index's validity, (2) systemic distortions in publishing and evaluation culture, and (3) proposed domains and criteria for competence-based assessment.

***Conceptual Framework Development:*** Two authors independently screened titles and abstracts, retaining items that (1) provided empirical data or legal analysis on recruitment, promotion, or training of physicians or (2) reported outcomes of workforce or educational reforms. Disagreements were resolved by consensus. The final narrative synthesis prioritized systematic reviews, comparative studies, and high-impact commentary, while illustrative case reports and investigative journalism were included when offering unique contextual detail. Based on this synthesis, we developed a six-domain meritocratic model to guide professional evaluation: clinical competence, teaching excellence, research quality, innovation and impact, ethical conduct, and leadership. This framework is designed as a flexible yet rigorous alternative to metrics-driven advancement, suitable for implementation across both academic and clinical career pathways.

### Anonymity and bias-mitigation safeguards (practical protocol)

- *Dossier standardization and redaction*: Candidates submit a structured file including indicator tables (risk-adjusted where applicable), de-identified outputs, and narrative statements without names, affiliations, grant numbers, or self-referential cues. A dedicated data office assigns each submission a pseudonymous identifier (ID).
- *Double-anonymous first pass: Two* reviewers from different regions score only the standardized indicators and redacted narratives using a fixed rubric (MPVS/IIS kernels) with 0–100 peer-normalized scales; free-text is structured to minimize identity leakage.
- *COI screens*: Automated COI checks (co-authorship networks, shared grants/employers) **+** COPE-based declarations; any positive COI → recusal and replacement (37).
- *Calibration and second pass*: A third reviewer (external region) adjudicates discrepancies >10 points. Only after convergence are identities unmasked for fit-for-post checks (teaching needs, service roles).
- *Decision governance*: Committee records decision logs, publishes aggregate dashboards (by specialty/region), and runs bias diagnostics (e.g., status-bias tests derived from double- vs. single-blind evidence) (38).
- *Audit and learning*. Annual audits of scoring variance, COI compliance, and outcome equity; protocols updated accordingly.

## Results

***Erosion of Educational Standards:*** Italy's primary and secondary school system has long struggled with outdated curricula and variable quality, as evidenced by national (INVALSI) and international (PISA) assessments (31, 39, 40). The rise of private institutions with minimal entry standards has allowed an unchecked expansion of diplomas. Subsequently, large numbers of students enter university—often including medical faculties—without solid academic foundations, further straining an overstretched admissions system (22–26, 33) (Figure 1).

***Becoming a Medical Doctor in Italy:*** Although tuition fees are relatively low, medical education entails a lengthy trajectory, including mandatory national-licensing examinations, competitive residency slots, and—in principle—structured mentorship (41–43). In practice, however, newly graduated doctors face limited access to high-quality residency training, political interference in hospital staffing, and insufficient recognition of advanced competencies (9, 15–25). Unsurprisingly, many talented practitioners look abroad, where transparent assessment and career progression are perceived to be more attainable.

***Draining Talent: The Emigration of Medical Graduates*** Italy's inability to retain its physicians has reached critical proportions: By 2021, over 11, 000 Italian doctors were officially employed in 21 OECD member states, with Germany and France hosting sizable contingents (22–24). Multiple studies highlight the multifactorial drivers behind this trend, including the absence of standardized specialty programs, perceived unfairness in promotions, and inadequate compensation (15–29, 44). In a cross-sectional survey of 307 Italian medical students, more than half expressed the intention to migrate post-graduation (45). The most cited reasons included the desire for better training opportunities, improved working conditions, and a more merit-based professional environment (46–49).

### Non-meritocratic practices in academic medicine

*Nepotism and favoritism*: Extensive investigations indicate that nepotism and favoritism remain systemic in Italy's academic environment (9, 22–29). The medical sector is not spared: Clusters of faculty with the same surname, or intertwined family networks, point to the preferential hiring of relatives or protégés (50–55). This entrenched system undermines open competition, blocking capable early-career clinicians and researchers.

*Gender disparities*: Gender bias further compounds these challenges. Although women represent a large proportion of medical students, they remain under-represented in senior academic positions and leadership roles (35, 56). In forensic medicine, for instance, fewer than 20% of residency programs are led by women—despite women making up a high percentage of graduates (35, 56). Such practices collectively degrade the merit principle, affecting both clinical outcomes and research productivity (53–55, 57–59).

***A Shifting Concept of Merit:*** Merit implies equitable opportunities and objective comparisons of competence (60–62). However, entrenched networks often overshadow formal qualifications: Senior healthcare managers in Italian hospitals—who control multimillion-euro budgets—are frequently handpicked for political loyalty rather than evidence-based expertise (63–68). This approach trickles down to department-director appointments, many of which bypass public scrutiny or data-driven assessments. In a parallel context, certain institutions have replaced competitive exams for high-level positions with so-called “career development” pathways, which may rely on experiential or subjective appraisals lacking transparency (69–72). Such practices foster mediocrity and perpetuate mistrust in the selection process.

***Identifying Leaders Without Disqualifying Followers:*** True leadership in medicine goes beyond issuing commands; it involves fostering trust, encouraging initiative, and balancing tradition with innovation. Emotional intelligence—allowing for constructive conflict resolution and collaborative decision-making—is the key to nurturing talent (73). However, some healthcare systems currently appear to favor “followers” with deep familiarity in regulations who do not challenge the chain of command (73). This expedites administrative workflow but risks stifling innovation.

The growing influence of artificial intelligence (AI) may further widen gaps between top achievers and those with limited resources or specialized training. Although AI holds the potential to level the playing field, in practice, sophisticated technologies often benefit those already positioned to exploit them (74).

***Variations in Medical Selection Systems:*** Across high-income democracies, physician recruitment follows two main tracks: cooptation, in which senior professionals directly select candidates, and open competition via examinations and professional portfolios (75–77). Cooptation can foster nepotism if unchecked, while public competitions may fail when they rely on superficial metrics (e.g., publication counts without quality appraisal). Italy exemplifies these pitfalls, with “concorsi” for hospital roles sometimes reduced to rote formalities and academic positions hindered by predetermined outcomes (78–82).

### Context: the Italian example

*Hospital Setting:* Legislative Decree No. 502/1992 and Decree No. 171/2016—together with related presidential regulations—establish that the appointment of attending and senior physicians in Italy must be conducted through public competitions (83, 84). However, these procedures often disproportionately emphasize publication metrics, such as raw citation counts, over measures of clinical competence, managerial capability, or innovation (78–80). This distortion becomes most visible in the selection of Complex Unit Directors (Direttori di Struttura Complessa), where regional political pressures or insider arrangements can eclipse objectively demonstrated qualifications (81, 82).

The appointment of General Directors of Local Health Authorities is likewise governed by Decree 502/1992 (as amended) and Decree 171/2016 (83, 84). Regional administrations must select appointees from a National List of Qualified Candidates, defined by a Prime Ministerial Decree of December 12, 2019, which outlines educational requirements, managerial experience, and evaluation methods (85). Although regions may conduct supplemental interviews or tailor decisions to local health objectives, they are prohibited from appointing individuals outside this roster. Despite such legal guardrails, the broad and loosely defined selection criteria create opportunities for political cooptation, where party loyalty may take precedence over merit-based competence.

*Academic Setting:* Academic career advancement is formally regulated through the Abilitazione Scientifica Nazionale (ASN)—a national habilitation process based on bibliometric thresholds such as H-index, impact factor averages, and publication quantity. However, substantial local discretion remains in the subsequent hiring phase. In numerous cases, informal panels composed of incumbent full professors preselect preferred candidates prior to open calls, thereby neutralizing competition and undermining procedural transparency (86–88).

*Limitations of Traditional Merit Indicators:* For decades, Italian nepotism has relied on two mutually reinforcing tactics (10–17, 34): First, job profiles are engineered to fit a pre-selected candidate rather than actual institutional needs. Second, commissioners are drawn by lot from the national professoriate—an ostensible anti-corruption safeguard that, in reality, masks a corrupt system. Should a randomly chosen commissioner contest a predetermined outcome, they are barred from nominating future candidates, ensuring widespread compliance (29, 30, 35, 47–55, 57–60, 89–109). The result is competition in name only: transparency focuses on the “drawing of balls,” while the balls themselves are pre-loaded.

*Unverified publications:* Candidates inflate CVs with guest authorships, honorary co-authorships, articles placed in high-impact journals via mutual-aid committees (110–112), or papers in predatory outlets that escape scrutiny. Current metrics fail to adjust for:

° Institutional asymmetries (e.g., private clinic owners listed on every subordinate's paper).
° Department heads whose names appear on all unit publications.
° Multi-center studies where local investigators gain authorship simply by contributing data (58, 59, 113).

Moreover, bibliometric tools ignore whether a work is truly innovative or merely narrative or meta-analytical.

*Patchy clinical documentation*: Comparative performance metrics (surgical outcomes, complication rates, and readmissions) are not systematically recorded, making objective assessment of clinical skill difficult (114–119).

*Superficial continuing medical education (ECM)*: ECM in Italy risks becoming a formality: Many courses are sponsored by pharmaceutical groups and offered free through societies, introducing conflicts of interest and little validation of competencies (15–18).

*Absence of multi-source feedback*: Peer, resident, and patient evaluations—crucial for measuring teaching quality and interpersonal effectiveness—remain rare in formal assessments (6, 13, 15–21, 34).

Collectively, these weaknesses allow patronage to eclipse performance, perpetuating a culture in which political alignment and social networks outweigh clinical excellence and scientific innovation.

### Beyond traditional approaches: potential solutions

*Anonymous, documented evaluation*: All applicants should submit digital portfolios that are cryptographically signed and independently audited, allowing selection committees to verify clinical data and publication authenticity (116–119).

*Revised scoring systems*. A composite MPVS could integrate five verifiable domains—clinical proficiency, complication rates, peer-reviewed research, educational contributions, and managerial innovation—while an IIS would measure real-world adoption of new procedures (116–120). Incorporating external frameworks such as the Stanford Top 2 % Scientists Ranking (121, 122), or an expanded IIS (121, 122), would reward substantive contributions rather than sheer publication volume. Digitized, verified curricula coupled with randomized, anonymized panels—including candidates from outside the national academy and from international programs—would further dilute the influence of entrenched “cordate” (interest groups) (Figure 7).

*Transparent commissioning and monitoring:* Rotating, blinded selection boards, and full public release of scoring sheets would curb nepotism and limit the scope for “pre-chosen” winners (73, 123–125).

*Concrete penalties for fraud*: Immediate disqualification and notification of professional bodies for falsified experience or publications would provide a strong deterrent (123, 126).

*Independent oversight*: Agencies with anti-corruption mandates should routinely compare declared competencies with post-appointment performance to ensure alignment (123, 126).

***Publication Metric Inflation:*** The H-index was once hailed as an objective yardstick of scholarly impact (50, 127), but inflated co-authorship, citation trading, and predatory publishing have eroded its credibility (128–131). Even reputable journals can be co-opted by collusive networks—often linked to commercial interests—whose influence has grown alongside private-equity acquisitions of healthcare centers. In Italy, “publication factories” enable favored candidates to amass impressive bibliographies devoid of meaningful contributions (50, 128–131).

Promoting meritocratic standards in medical career advancement—using Italy as a case study—requires a focused approach that addresses health policy, academic structures, equity in professional pathways, and regional disparities in medical career trajectories. Such efforts must also embrace institutional critique and welcome reform-oriented, comparative governance, and education frameworks. An analysis of the past two decades reveals a sharp rise in medical publications and H-index accumulation, often driven by opportunistic publication strategies. This trend has resulted in a disproportionate ratio between publication volume and true scientific progress, with limited correlation to meaningful discoveries or advances in medical disciplines (50, 132).

**Figure 2:**
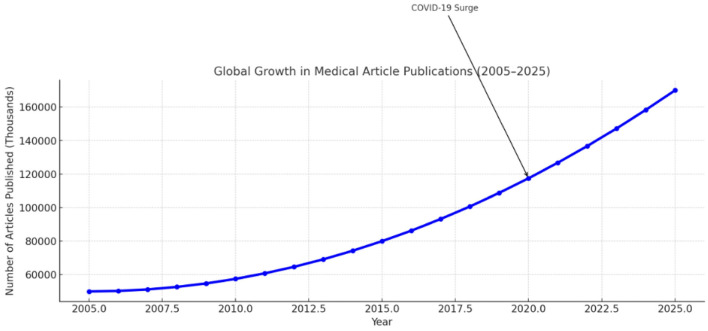
Global Growth medical publications in the last 20 years.

**Figure 3:**
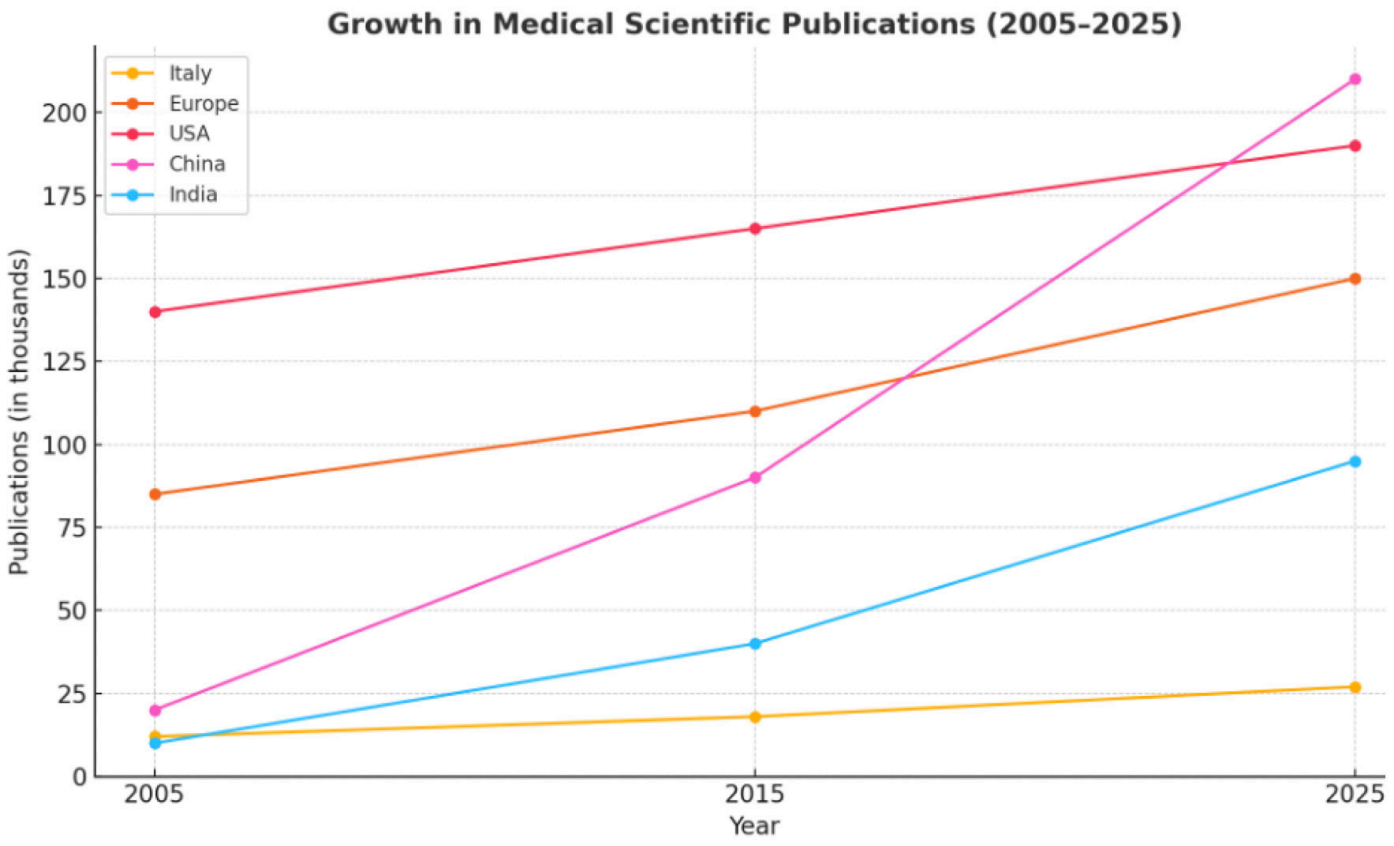
Trend of Medical publications growth (%) in the last 20 years divided for 5 different regions.

**Figure 4:**
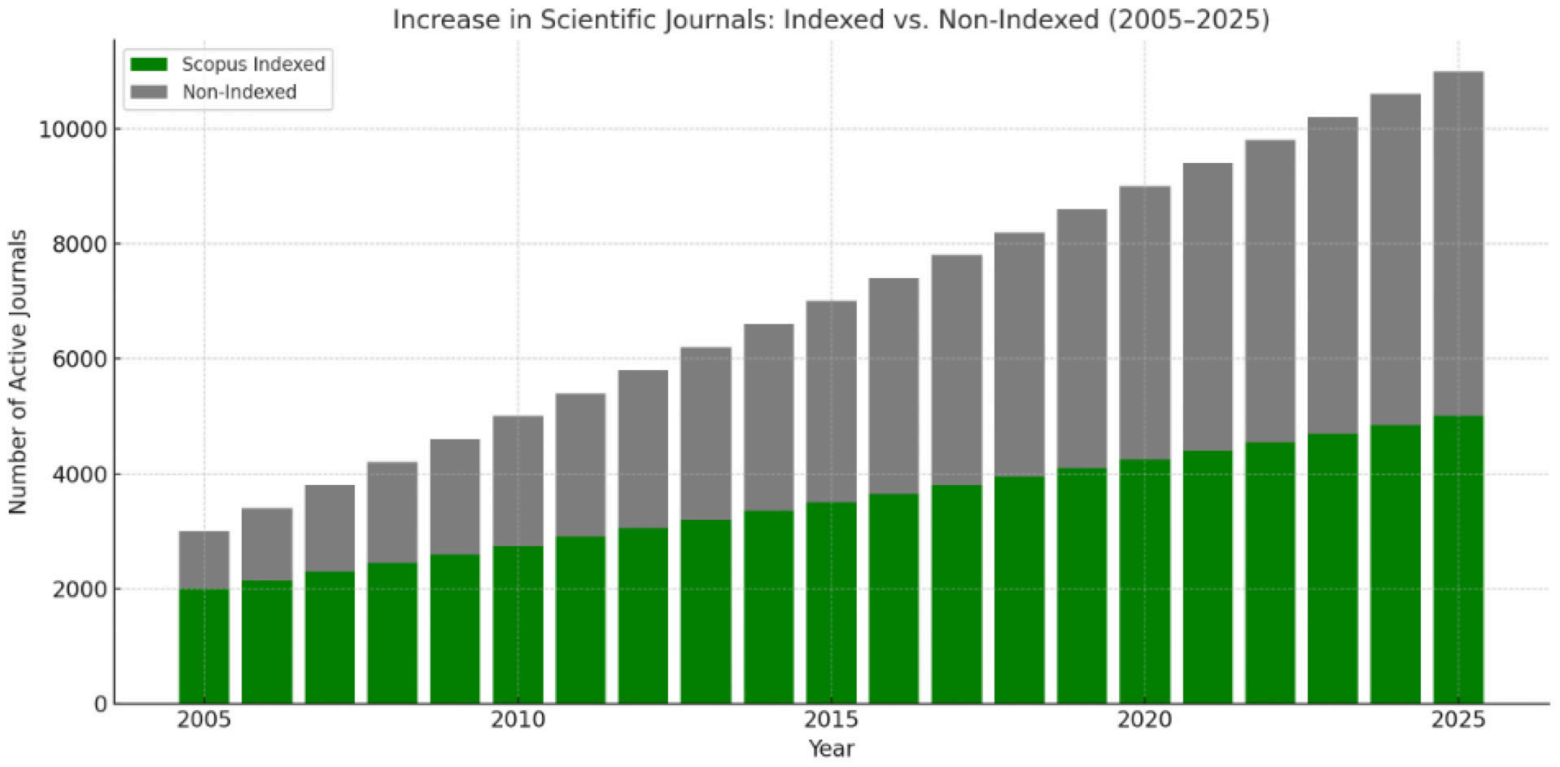
Scientific journals growth in the last 20 years here divided for indexed and non-indexed.

*Increase in Medical Article Publications (2005–2025):* Over the past 20 years, the H-index—a metric reflecting both productivity and citation impact—has markedly increased among academic medical professionals in Italy, Europe, USA, China, and India (50). Simultaneously, the global number of medical articles published has surged, propelled by advances in technology, the rise of open-access publishing, and increased research investment from emerging economies (50, 132) (Figures 2–4; Table 1).

**Table 1:** Growth (%) of Medical publications in the last 20 years divided for 5 different regions with notable drivers.

**Region**	**Publications in 2005**	**Publications in 2025**	**Growth (%)**	**Notable Drivers**
Italy	12,000	27,000	+125%	Steady growth driven by national academic incentives (e.g., *Abilitazione Scientifica Nazionale*), increased collaboration with EU-funded projects, and focus on bibliometric-driven career advancement (163, 164).
Europe	85,000	150,000	+76%	Europe (excluding Italy) Benefited from cross-border collaboration, Horizon Europe funding, and open-access mandates. Strong academic infrastructure and inter-country networks supported consistent publication output (165).
USA	140,000	190,000	+36%	Already a leader in 2005, the USA saw more modest relative growth but still leads in absolute output. NIH funding, institutional publication pressure, and advanced research ecosystems contribute to sustained productivity (166, 167).
China	20,000	210,000	+950%	A phenomenal rise driven by massive government investment in science and technology, performance-based academic incentives, and a national push for international journal publications (168).
India	10,000	95,000	+850%	Growth accelerated in the 2010s with improved research funding, private medical institutions' expansion, and government research initiatives (e.g., ICMR, DBT). Emphasis on open-access journals also played a role (169).

Notable spikes in publication volume have occurred in response to global health emergencies such as the COVID-19 pandemic (133, 134) and through the adoption of AI-based research techniques (134–136). Between 2005 and 2020, PubMed-indexed biomedical publications nearly doubled (135). During the peak of the COVID-19 crisis (2020–2021), publication rates rose sharply, especially in infectious disease, epidemiology, and public health domains (133, 135). Since 2018, medical research utilizing AI and machine learning (ML) has increasingly populated high-impact journals (136).

*Increase in Scientific Journals (Indexed vs. Non-Indexed, 2005–2025*): The global number of active scientific journals has increased significantly over the past two decades, driven by the rise of open-access publishing and digital dissemination models (137–141). Notably, Scopus- and Scimago-indexed journals have expanded steadily, particularly in the fields of biomedicine, engineering, and computer science (139–142) (Figure 2; Table 1). In contrast, non-indexed and predatory journals have proliferated more rapidly, especially in developing regions and through unregulated platforms (141–143). This growth has raised substantial concerns regarding editorial standards, peer-review integrity, and market manipulation. The academic community increasingly relies on trusted indexing platforms to discern the credibility of journals (139–142).

**Figure 5:**
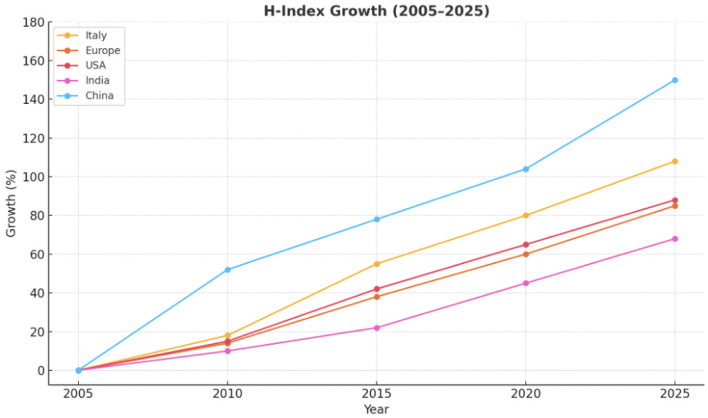
Trends of H-index growth in 5 different global regions in the last 20 years.

***The H-index*** is intended to capture the scientific contribution of individual researchers, and its steady rise over the past two decades across all examined regions underscores a broader transformation in research productivity, scholarly collaboration, and the metrics used for academic evaluation (Figure 5).

**Figure 6:**
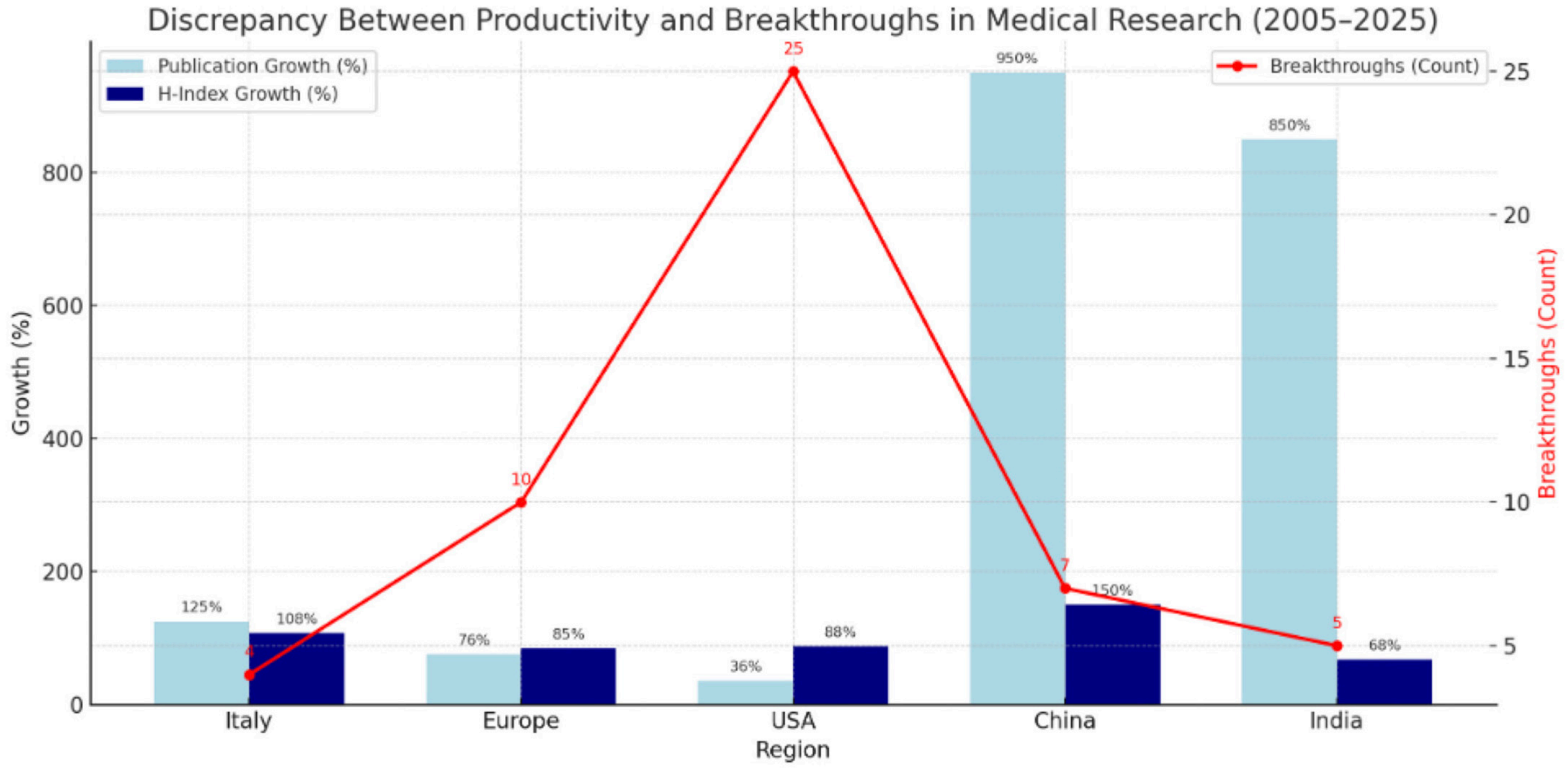
We established the comparison between the trends of H-index and Publications, and breakthroughs in 5 different regions. Trends are remarking the dissociation between publications/H-index and scientific innovation.

The accompanying infographic offers a visual synthesis of the growing disparity between quantitative scholarly productivity and qualitative scientific innovation across major regions from 2005 to 2025. Quantitative metrics such as the number of publications and the H-index (represented by the blue and dark blue bars, respectively) have risen sharply, especially in countries like China and India. In contrast, the red line—indicating breakthrough-level scientific contributions, such as paradigm-shifting studies identified through expert consensus and Stanford rankings—has shown only modest growth (Figure 6).

This divergence underscores a critical tension in modern research evaluation: Increased output does not necessarily equate to increased impact (50, 132, 134). China and India demonstrate exponential growth in publication volume and citation metrics but produce comparatively fewer transformative innovations (112). Meanwhile, the United States and Western Europe maintain leadership in high-impact research despite relatively stable publication volumes. Italy exhibits a middle-ground trend—moderate increases in productivity accompanied by a smaller but meaningful number of standout contributions (9, 144).

The visualization reinforces growing concerns that citation-based metrics alone (e.g., the H-index) are insufficient proxies for innovation or clinical relevance (7). Experts increasingly advocate for evaluative frameworks that distinguish between volume-based productivity and genuine advancement of medical science (9, 50, 112, 115, 132, 134, 144, 145) (Figure 6).

The accompanying infographic illustrates the trajectory of scholarly productivity in reproductive medicine in Italy from 2005 to 2025, as measured by H-index growth and the volume of scientific publications. Over the past two decades, a substantial increase is evident in both metrics, reflecting enhanced research output and citation impact among Italian reproductive health professionals (50, 131). This upward trend is corroborated by international bibliometric analyses and national journal indexing databases (134, 142).

**Figure 7:**
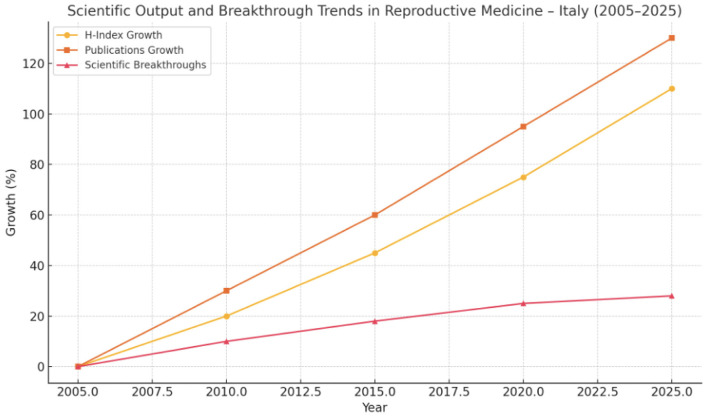
We established the comparison between the trends of H-index and Publications, and breakthroughs in one specific medical field—reproductive medicine—in Italy.

However, the pace of truly transformative innovations—defined as clinical breakthroughs that meaningfully alter standards of care—has remained relatively modest (Figures 6, 7) (9, 146). Despite the increasing publication volume, concerns persist regarding the reproducibility and translational impact of much of this research (145). Emerging tools such as AI-driven literature analysis also question the real contribution of volume-based metrics to clinical advancement (134). This growing divergence highlights a central tension in modern academia: Quantitative bibliometric expansion does not necessarily correspond to qualitative progress in patient care.

*Toward an Italian Implementation:* Introducing external verification tools—such as the *Stanford Top 2% Scientists Ranking*—and robust IIS measures would help prioritize substantive contributions over sheer publication volume (121, 147). Digitizing CVs and anonymizing evaluation panels may reduce the influence of local patronage networks (121, 147–150). At the same time, legislative reforms should retain meaningful penalties for “abuso d'ufficio,” thus maintaining deterrence against academic or institutional collusion (132, 141, 143).

*Rising Concerns: Legal Reforms and Diminished Accountability* Recent proposals to narrow the legal definition of “abuso d'ufficio” risk weakening safeguards against fraudulent appointments (132, 141, 143, 151–153). Critics warn that such changes could foster an environment where favoritism and nepotism flourish with reduced fear of legal consequences.

**Figure 8:**
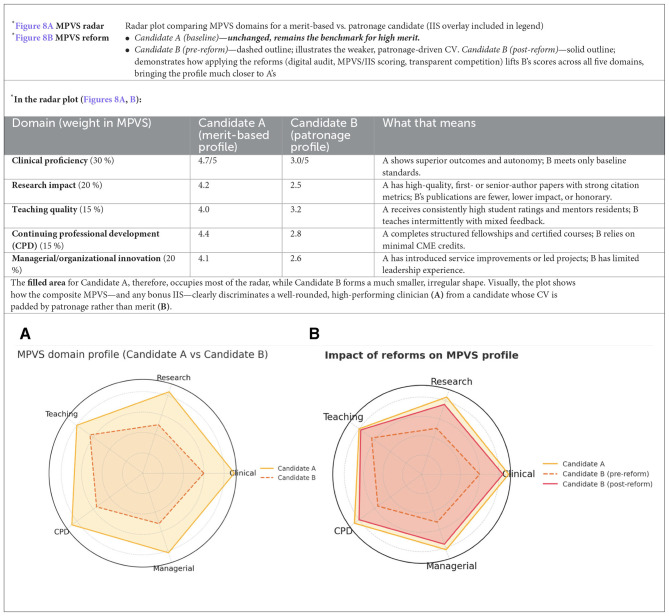
**(A, B)** Radar plot comparing MPVS domains for a merit-based vs. patronage candidate.

**Figure 9:**
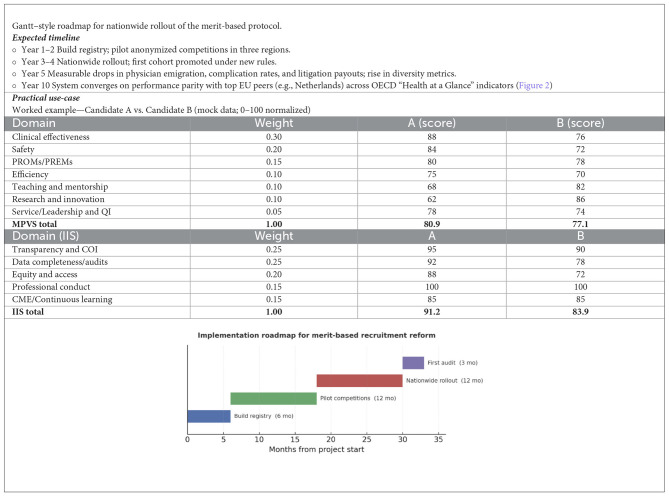
Roadmap.

*Proposal for a Merit-Based Assessment Protocol:* We propose an updated framework for selection, recruitment, and career advancement of medical personnel in healthcare, hospital, and academic settings. The aim is to replace discretionary, co-optative practices with transparent, competitive, merit-based criteria (see Tables 2–5; Figures 8A, B, 9).

**Table 2:** Core data requirements.

**Domain**	**Mandatory documentation**	**Linked table**
Clinical and surgical activity	Outcomes, complication rates, and adherence to gold-standard protocols, extracted from the patient EMR	Tables 1-4a
Research performance	Publication type, journal ranking, H-index, and evidence that findings changed clinical practice	Tables 1-4b
Teaching performance	Course load, student evaluations, and pass rates	Tables 1-4c
Professional behaviors	Annual scores for leadership, teamwork, empathy, and reputation, based on a validated tripartite survey	Tables 1-4d
**Clinical and surgical activity**									
**#**	**Date**	**Performancevenue**	**Performance**	**Performance type**	**Difficulty level**	**Expected vs.achieved result**	**Major complications**	**Minor Complications**	**Legal disputes/convictions**
									
**Research performance**									
**#**	**Date**	**Rsearch venue**	**Study type:** ***original RCT, retrospective, review, systematic review, systematic review with meta-analysis***.	**Publication journal**	**Difficulty level (impact score)**	**Author position**	**Cross-check of author names**<**sup**>**2**<**/sup**>	**Discoveries that changed medical practice**	**Total original studies as first author**
									
**Teaching performance**				
**Teaching subject**	**Date**	**Venue/institution**	**Average student rating**	**Pass rate (%)**
				
**Professional behaviors**				
**#**	**Venue institution**	**Leadership approach**	**Fellowship approach**	**Empathy**
				
**Table 1 Recording of training parameters of competence**.
**4a Work Experience** “Performance Venue” indicates where the procedure or clinical work took place (e.g., hospital and clinic). “Performance Type” can include surgery, consultation, and procedure. The “Difficulty Level” column is used to classify the complexity (e.g., low, moderate, high). In the “Expected vs. Achieved Result” column, outcome data is recorded. Significant complications and any legal issues in the respective columns are documented.
**4b Research** “Cross-Check of Author Names” refers to verifying consistency across multiple institutions or databases. The column, the exact type of research conducted and the corresponding publication details is recorded. “Difficulty Level (Impact Score)” can reflect the scope or rigor of the study. “Author Position” is the column to place the author list (e.g., first, last, corresponding). The “Discoveries That Changed Medical Practice” coulmn is used to highlight major breakthroughs.
**4c Teaching Skills** “Teaching Subject” indicates the course or topic taught (e.g., Anatomy, Internal Medicine). “Venue/Institution” is where teaching was delivered (university, hospital, conference, etc.). “Average Student Rating” should be the mean evaluation score from student feedback. “Pass Rate (%)” indicates the percentage of students successfully completing the course or exam.
**4d Ability to Interact with the Work Community** “Leadership Approach” may include examples of leading teams or initiatives. “Followership Approach” captures collaborative skills, openness to feedback, and willingness to support colleagues. “Empathy” refers to interpersonal sensitivity and patient-centered communication. “Reputation” can be assessed via peer reviews, supervisor feedback, or formal evaluations.

**Table 3:** Competence Identification Criteria.

**(a) Levels of clinical activity proficiency: this table defines a six-tier progression system for measuring clinical and procedural autonomy**		
**Level**	**Description**	**Note**
0	Observation only	No active involvement; early training phase
1	Assisted participation	Requires constant supervision
2	Independent execution of simple procedures	Demonstrates basic competence
3	Autonomy in moderate-complexity procedures	Acts independently with minimal supervision
4	Full autonomy in complex procedures	Indicates advanced specialization and direct responsibility
5	Mentorship and supervision of peers	Recognized as expert tutor/trainer
**(b) Weighted evaluation domains for merit-based assessment—this matrix assigns relative weights to five key domains for balanced performance evaluation**		
**Domain**	**Weight**	**Indicators**
Clinical and surgical competency	30%	Case volume, complexity, complications
Scientific output	20%	H-index, impact factor, weighted citation ratio (WCR)
Teaching and training quality	15%	Student feedback, hours taught, supervision
Professional development (CME)	15%	Accredited CME, fellowships, advanced training
**(c) Innovation scale: from local ideas to global standards—this scale provides a structured evaluation of innovation maturity, from conception to international impact**		
**Score**	**Definition**	**Evidence examples**
0–10	Conceptual idea, not yet tested	Early-stage papers, exploratory concepts
11–20	Pilot-tested in 1–2 institutions	Local implementation, experimental guidelines
21–30	Validated innovation with proven outcome benefits	Multicenter results, adoption in ≥3 centers
31–40	Integrated in widespread clinical practice	International guidelines, patent approval
41–50	Globally transformative and policy-defining	Referenced by WHO, established global gold standard

(a) Clinical and surgical competency is evaluated using documented evidence (hospital databases and electronic charts) of the level of autonomy achieved in each procedure. Each level corresponds to a score, which can be combined with additional outcome indicators. (b) The proposed MPVS includes five domains, each with a specific weight. Every domain encompasses verifiable parameters (audits, public databases and e-learning platforms).

**Table 4:** Intrinsic professional values measurement by the five more important domains: proposed indicator and H-index limitations.

**Evaluation domain**	**Proposed metric or indicator**	**Limitations of H-index in this domain**
Clinical Competence	Peer-reviewed case audits, clinical KPIs, complication rates	Does not reflect hands-on clinical skills or patient outcomes
Teaching Excellence	Student feedback, curriculum development, and peer observation	Ignores educational roles and quality of teaching
Research Quality	Intrinsic value of research, originality, reproducibility, and societal impact	Prioritizes citation count over substance or novelty
Innovation and Impact	Patent output, grant innovation score, and policy translation	Fails to capture creative, interdisciplinary, or translational outputs
Ethical and Professional Conduct	Ethics review outcomes, patient feedback, and conflict-of-interest transparency	Not sensitive to professional integrity or conduct
Leadership and Mentorship	Supervision outcomes, team feedback, and role in organizational development	No recognition of leadership or mentoring contributions

**Table 5:** System level impacts of merit-based reform.

**Dimension**	**Current weaknesses**	**Post-reform impact (mechanism → outcome)**
**Clinical quality**	Heterogeneous skill levels; complication and readmission data rarely audited	Transparent MPVS scoring ties promotions to audited outcomes → low-performing units trigger remedial action; high performers proliferate. International evidence links outcome-based credentialling to 10–15 % reductions in surgical complications within 3 years.
**Workforce retention and migration**	•>11,000 doctors abroad • Residency bottlenecks • Perception that “who you know” matters more than competence	Meritocratic contests + published score sheets rebuild trust; young doctors see a fair career ladder → projected 20–25% decline in annual emigration within 5 years. Returning expatriates gain competitive credit for validated overseas experience.
**Gender and diversity equity**	Women and internationally trained doctors under-represented in leadership	Anonymised, point-based evaluation removes name recognition bias; leadership eligibility depends on MPVS/IIS, not patronage → faster narrowing of gender gap (modeled at +4–5 % female unit directors per 5-year cycle).
**Innovation uptake**	Breakthroughs diffuse slowly; bibliometric incentives favor quantity over novelty	IIS rewards guideline adoption and patents, not raw citations → R&D budgets shift toward clinically transformative projects; time from publication to national guideline citation shortens.
**Cost-effectiveness**	Inefficient staffing; litigation costs from adverse events	Better match between competence and role reduces preventable complications and malpractice claims; estimated €350–450 million annual savings (≈ 1 % of Italy's hospital spend).
**Public trust and transparency**	“Concorsi truccati” scandals erode confidence	Open data registry, external audits, and automatic fraud penalties signal accountability → higher patient-satisfaction scores and better compliance with public-health initiatives.

## Competence composite metrics

### Clarification of evaluation metrics

a) *Weighted Citation Ratio (WCR):* A bibliometric index that adjusts raw citation count by factoring in the **impact factor (IF)** of the journal, the number of co-authors, and self-citation exclusion. It provides a more realistic estimate of an individual's scientific influence by penalizing inflated authorship and self-referencing behaviors (147).
b) *Impact Innovation Score (IIS):* An applied metric designed to evaluate whether a physician's work has led to real-world clinical implementation. While traditional bibliometrics reflect volume and visibility, the IIS verifies whether an innovation has been (a) adopted in national protocols; (b) included in clinical guidelines, or (c) established as a global standard (e.g., WHO endorsement and CE/FDA certification).

### Key elements

° **Digital performance registry:** Candidates upload clinical, educational, and research data to a secure platform with digital signatures and cross-checks in external databases (Table 1).
° **Anonymous evaluation:** Selection committees review fully de-identified dossiers, reducing bias toward “well-known” applicants.
° **Composite scoring:** The MPVS forms the baseline ranking; an IIS bonus rewards demonstrable innovation (Figure 8).
° **Monitoring and sanctions:** ANAC and the Court of Auditors audit every competition to identify “tailor-made” calls or false declarations and apply penalties.
° **Dynamic feedback loop:** Low-scoring units must submit corrective plans, with re-audit driving continuous quality improvement.
° **Reputation economics:** High aggregate MPVS attracts patients and top applicants, creating competitive pressure on lagging centers.
° **Policy agility:** Real-time registry analytics enable early detection of specialty shortages and adjustment of residency slots.

### Interpretation and decision rule

- **Candidate A:** MPVS 80.9 (Strong) + IIS 91.2 (Excellent) → **Promotion eligible now**; targeted development in research/mentorship.
- **Candidate B:** MPVS 77.1 (Strong) + IIS 83.9 (Strong) → **Eligible with conditions**; development plan on clinical outcomes/safety; maintain research excellence.
- **Equity guardrail:** if any IIS domain < 60 (e.g., data completeness), promotion is **deferred** pending remediation.

In sum, the reform shifts incentives from patronage to measurable performance, creating a virtuous cycle that improves patient outcomes, workforce stability, and fiscal sustainability. Our analysis illustrates the paradigm shift in how the H-index is perceived in the evaluation of medical career candidates. Once regarded as a robust and objective metric of academic merit, the H-index has seen a significant decline in its indicative value. This decline stems from several critical distortions in the current academic publishing landscape:

° The proliferation of publications co-authored without genuine contribution.
° The inflationary trend of author lists, often including dozens of names with limited relevance.
° The redundancy of articles recycling previously published concepts without adding innovation or original insight.
° The unchecked growth in the number of journals—indexed and non-indexed alike, predatory and non-predatory—contributing to volume over value.
° The corporatization of prestigious journals, whose editorial policies increasingly align with institutional and society-based lobbying rather than scientific rigor.
° The disproportionate emphasis on narrative reviews and consensus papers, often favored over original research with authentic methodological foundations.

Together, these dynamics erode the H-index's reliability as a proxy for scientific excellence and highlight the urgent need for more nuanced, qualitative, and innovation-sensitive criteria in merit-based academic and medical career advancement.

## Discussion

A merit-based system is indispensable for high-quality patient care and scientific progress. Yet, nepotism, distorted bibliometrics, and weak oversight still block the deserving candidates in Italy. *Digital verification, composite scoring, blinded review panels, and real penalties for fraud* can restore credibility without dismantling legitimate mentorship networks (121, 152–154).

Without such reforms, the emigration of skilled professionals will continue, widening the gap between politically favored “followers” and genuine innovators. A robust meritocracy—grounded in accountability, ethical rigor, and continuous evaluation—restores trust and aligns incentives with patient outcomes and scholarly impact (141, 143, 152).

### The legacy of a flawed system and the promise of reform under the new law

Until recently, entering the academic ranks in Italy involved navigating a two-stage process dominated by a central agency, ANVUR, composed of university professors. This agency established, at its discretion, the type and quantity of scientific output required to obtain the so-called National Scientific Qualification (ASN). Once obtained, this qualification allowed candidates to apply for open faculty positions at individual departments. However, hiring was effectively controlled by the departments themselves, often resulting in a nominal competition that functioned more as an internal selection—commonly referred to as a “chiamata” (invitation). A critical flaw in this system was that the ASN had no cap on the number of successful candidates. Since passing the qualification did not displace others, committees had little incentive to uphold high standards, and nearly all applicants were routinely granted the title—regardless of merit. Consequently, thousands of hopefuls obtained the qualification, believing that it would guarantee an academic appointment. In reality, appointments were frequently awarded not on merit but through personal ties with departments, where many candidates had studied or worked previously. The outcome was a proliferation of academic inbreeding and localism, which severely limited intellectual mobility and undermining fairness. This environment fostered a culture where career progression became decoupled from merit, and the national academic landscape stagnated under the weight of patronage networks. Entire careers were built within the same institution, shutting out the possibility of knowledge circulation that had once enriched even the most peripheral universities across Italy. In May 2025, new legislation proposed by Minister Anna Maria Bernini marked a significant shift W. The National Scientific Qualification was abolished, and ANVUR's powers were scaled back (132, 155). Recruitment is currently tied directly to open positions within individual universities. Anyone meeting the Ministry's requirements can apply, with documentation self-certified. Evaluation is handled by a five-member committee: one internal member from the hiring university and four drawn randomly from a national pool (141, 143, 152). Despite this progress in simplifying procedures and aligning selection more closely with actual needs, serious doubts remain about the impartiality and meritocratic quality of the new system. Randomly selected external evaluators still belong to academic subfields tightly governed by internal networks, where pre-established decisions and unwritten agreements can continue to influence outcomes. As a result, the reform may correct some inefficiencies without truly addressing the structural deficit of meritocracy in Italian academia (121, 122, 147, 156).

Currently, the recruitment of competent personneland the promotion of medical careers remain vulnerable to lobbying pressures, especially in large public systems. Lax scrutiny of publications, clinical logs, and teaching records still rewards “predestined” candidates linked to power networks (90, 91).

We introduce the *Merit-based Professional Value Score (MPVS)* and the *Integrity and Impact Score (IIS)* as complementary tools for merit-based progression. MPVS captures risk-adjusted clinical value, education, and innovation, whereas IIS covers transparency, data integrity, equity, conduct, and continuous learning.

Embedding objective indicators (MPVS, IIS, and WCR) within transparent, anonymous selection procedures offers a realistic path to defend meritocracy and maintain high standards of care. The accompanying tables (Tables 1, 6, 7) provide a modular framework that can be updated and adapted internationally, provided it is paired with vigilant oversight and protections for whistle-blowers.

**Table 6:** Growth (%) of Medical Journals in the last 20 years divided for 5 different regions with notable drivers.

**Region**	**Journals in 2005**	**Journals in 2025**	**Growth (%)**	**Notable trends**
Italy	~45	~120	+167%	Many journals launched to support national academic evaluation systems [e.g., Agenzia nazionale di valutazione del sistema universitario e della ricerca (ANVUR)], including bilingual or regionally focused ones. However, indexation in Scopus or PubMed remains a barrier for some (170).
Europe	~800	~1,400	+75%	**Europe (excluding Italy)** European scientific publishing has diversified with strong contributions from Germany, the Netherlands, Spain, and Scandinavian countries. Many traditional societies digitized older journals and launched OA counterparts (171).
USA	~1,000	~1,300	+30%	**United States** Home to many of the world's most influential journals (e.g., *NEJM, JAMA*), the US saw modest relative growth but remained dominant in high-impact publishing. New journals mostly emerged in niche or interdisciplinary fields (172).
China	~100	~750	+650%	**China** Massive expansion driven by government-supported research incentives and academic publishing mandates. Many are currently indexed in Scopus and Medline, especially through English-language editions or co-publication with global publishers (173).
India	~80	~550	+587%	**India** Proliferation of institutional and society-based journals—especially in open-access formats. While the volume is high, issues around peer review rigor and impact factor persist (174).

**Table 7:** Four different model criteria to assess medical competence in medical career progression.

**Country/model**	**Evaluation criteria**	**Citations**
**Netherlands (UMCs)**	Audits of clinical workload, complication rates, and validated teaching performance	(175, 176)
**Canada (Tenure Track)**	Double-blind external reviews focused on knowledge translation indicators	(177, 178)
**UK (NHS Clinical Excellence Awards)**	Transparent clinical and academic portfolios regulated by General Medical Council (GMC)	(179, 180)
**Sweden (Karolinska Institutet)**	Detailed logs of clinical activity and proof of publication originality	(148, 149)

A radical overhaul of the criteria and metrics used to measure merit in physician recruitment is urgently required; without it, the reputation and efficiency of the entire healthcare system will continue to deteriorate.

*Political economy and the public–private* mix Italy's Servizio Sanitario Nazionale (SSN) is a regionally governed, tax-funded system that guarantees universal coverage. Service delivery combines public providers with a substantial network of accredited private facilities, with notable inter-regional variation in service mix and capacity. Recent syntheses by the OECD and the European Observatory highlight this mixed delivery model and persistent territorial heterogeneity in healthcare access and performance. Our merit-based progression proposal is designed to be policy-agnostic to that mix, while remaining performance-anchored through common indicators and transparent reporting (157).

Ongoing reforms—particularly PNRR Missione 6 and the implementing DM 77/2022—envisage stronger primary/community care via *Case della Comunità* and related territorial standards. These reforms reshape care pathways and data flows that are essential to measuring outcomes fairly across organizations. We explicitly require that any adoption of our merit framework mandates (i) interoperable clinical and administrative datasets across public and accredited-private providers, (ii) uniform indicator definitions and risk-adjustment, and (iii) public dashboards at regional and facility level to preserve accountability (158).

Because financing choices influence incentives for clinicians and providers, we also note system-level parameters relevant to merit implementation (e.g., out-of-pocket shares above the OECD average; regional procurement and contracts). To prevent the emergence of a “two-track” labor market that might privilege certain provider types, we include four safeguards: (1) one national merit rubric binding for all accredited employers; (2) portability of merit credits across regions/providers; (3) outcome-weighted incentives tied to transparent, risk-adjusted indicators; and (4) equity screens (monitoring case-mix, waiting-time differentials, and complication rates by socioeconomic status). These safeguards keep progression comparable and equitable, regardless of the relative expansion of public or accredited-private provision (159).

*References for this subsection (examples):* OECD/European Observatory Italy Country Health Profile 2023; European Observatory Italy: Health System Review 2022; AGENAS/PNRR Missione 6 and DM 77/2022 implementation documents on *Case della Comunità* (160).

### Limitations

Generalizability beyond medicine. Our flow chart and metrics are optimized for the medical context (e.g., risk-adjusted clinical outcomes, safety, and PROMs). To align with Italy's single academic career architecture (Law 240/2010; **ASN** as an enabling prerequisite across all faculties), we propose a two-layer architecture: (i) a cross-faculty core (research quality/impact, teaching and mentorship, service/leadership, data integrity, equity, and professional conduct) with unified definitions and peer normalization; and (ii) discipline adapters that replace clinical-specific indicators with domain-appropriate ones (e.g., monographs/editions in the humanities; patents, prototypes, or design deliverables in engineering/arts), preserving comparability at the core while maintaining validity within each settore concorsuale/SSD. Future work should co-design these adapters with ANVUR/ASN stakeholders and test inter-faculty portability (161).

*On anonymity*. Given the small size and topic transparency of many SSDs, complete de-identification is rarely feasible. We should therefore adopt functional anonymization (double-anonymous scoring of standardized and redacted dossiers) combined with COI/recusal rules, cross-regional external reviews, and bias audits (status-bias diagnostics and equity screens). Randomized and observational evidence indicates that double-anonymous review reduces institutional/status bias, though it is not a panacea—hence the need for layered safeguards and public decision logs (162, 181–187).
